# Design, Fabrication, Simulation and Characterization of a Novel Dual-Sided Microelectrode Array for Deep Brain Recording and Stimulation

**DOI:** 10.3390/s16060880

**Published:** 2016-06-15

**Authors:** Zongya Zhao, Ruxue Gong, Hongen Huang, Jue Wang

**Affiliations:** 1 The Key Laboratory of Biomedical Information Engineering of Ministry of Education, Institute of Biomedical Engineering, School of Life Science and Technology, Xi’an Jiaotong University, Xi’an 710049, China; zhaozongya2010@stu.xjtu.edu.cn (Z.Z.); gongruxue@stu.xjtu.edu.cn (R.G.); huanghongen@stu.xjtu.edu.cn (H.H.); 2 National Engineering Research Center of Health Care and Medical Devices, Xi’an Jiaotong University Branch, Xi’an 710049, China

**Keywords:** Parkinson’s disease, mechanisms of deep brain stimulation, dual-sided microelectrode array

## Abstract

In this paper, a novel dual-sided microelectrode array is specially designed and fabricated for a rat Parkinson’s disease (PD) model to study the mechanisms of deep brain stimulation (DBS). The fabricated microelectrode array can stimulate the subthalamic nucleus and simultaneously record electrophysiological information from multiple nuclei of the basal ganglia system. The fabricated microelectrode array has a long shaft of 9 mm and each planar surface is equipped with three stimulating sites (diameter of 100 μm), seven electrophysiological recording sites (diameter of 20 μm) and four sites with diameter of 50 μm used for neurotransmitter measurements in future work. The performances of the fabricated microelectrode array were characterized by scanning electron microscopy (SEM), electrochemical impedance spectroscopy (EIS) and cyclic voltammetry. In addition, the stimulating effects of the fabricated microelectrode were evaluated by finite element modeling (FEM). Preliminary animal experiments demonstrated that the designed microelectrode arrays can record spontaneous discharge signals from the striatum, the subthalamic nucleus and the globus pallidus interna. The designed and fabricated microelectrode arrays provide a powerful research tool for studying the mechanisms of DBS in rat PD models.

## 1. Introduction

Parkinson’s disease (PD) is a degenerative disease with cardinal motor symptoms that correlate with the loss of dopaminergic neurons in the substantia nigra of the brain. PD is characterized by akinesia, bradykinesia, rigidity, resting tremors, and other motor and postural impairments [1,2]. Although deep brain stimulation (DBS) of the subthalamic nucleus (STN) has proved to be an effective symptomatic treatment in advanced PD for well selected patients [3,4,5,6,7,8], its mechanisms of action are still not well understood, which will limit further clinical application of DBS.

Currently, investigations of the mechanisms underlying DBS have attracted great interest in the scientific community. On the one hand, although numerous experimental studies have analyzed the effect of DBS at the single cell level using *in vivo* microelectrode recording in recent years [9,10,11,12,13,14,15], the tools used by these researchers were based on wire electrodes. On the other hand, recent studies have shown that the mechanisms underlying DBS correlate with the regulation of basal ganglia system (BGS) which consists of four interconnected nuclei: the subthalamic nucleus (STN), globus pallidus (futher divided in pars interna, GPi, and pars externa, GPe), substantia nigra (futher divided in pars compacta, SNc, and pars reticulata, SNr) and striatum [16,17,18,19]. Therefore, the mechanisms underlying DBS could be better understood from the perspective of functional network interactions among different nuclei of BGS [20], which need to simultaneously obtain electrophysiological information from multiple nuclei of BGS during electrical stimulation of STN. Although wire electrodes could be used to record neuronal discharge signals from multiple nuclei, for example, Shi *et al.* implanted eight stainless steel teflon-insulated microwires into the striatum, globus pallidus and SNr to record their neuronal discharge signals simultaneously [21]. However, in this case, multiple wire electrodes need to be implanted into different brain nuclei, and in addition, another type of wire electrode should be implanted into STN for electrical stimulation, which will greatly increase the complexity and difficulty of surgical procedures. Moreover, the limitations of wire electrodes, such as difficult implantation, low spatial resolution, low reproducibility and manual construction are also well known [22,23]. Therefore, in order to better study the mechanisms of DBS, a multifunctional microelectrode which can stimulate STN and obtain electrophysiological information from multiple nuclei of BGS is urgently needed.

The rapid development of micro-electromechanical systems (MEMS) technology in the biomedical field provides a feasible solution for the above-mentioned problems. With MEMS technology, the precise definition of electrode size and shape can be realized, and multiple recording/stimulation sites can be fabricated on a single probe shank [24,25,26]. Since the pioneering work of Wise *et al.* [27], a growing number of silicon-based neural probes have been developed, with a variety of electrode geometries and methods for fabrication and assembly, including the well-known Michigan probes [28,29] and Utah electrodes [30,31,32]. With the development of neuroscience, especially brain science, novel microelectrode arrays are urgently needed by neuroscientists to further understand the working principles of the nervous system. On the one hand, the performances of MEMS microelectrode arrays have been improved in many aspects [33], e.g., three-dimensional arrays [34], dual-sided microelectrode arrays [35,36], microelectrode arrays with high-density stimulation/recording sites [37], integrated electronics or microfluidic channels [38,39,40,41,42,43], silicon probes for optogenetics or infrared stimulation probes [44,45]. On the other hand, according to different experimental requirements, some specially designed microelectrode arrays have been developed. For example, Xu *et al.* [46] designed and fabricated a multi-shank electrode which can reach multiple regions of the hippocampus simultaneously according to the special neuro-anatomical structure of the hippocampus. Márton *et al.* [47] designed and characterized a microelectrode array system specifically for obtaining *in vivo* extracellular recordings from three deep-brain areas of freely moving rats simultaneously. Furthermore, rats have become a common animal model for studying the mechanisms of DBS due to their low cost and being easy to model [20,48]. However, to the best of our knowledge, there is no report about a specially designed dual-sided microelectrode array which is used for studying the mechanisms of DBS in a rat PD model, can stimulate STN and record electrophysiological information of the striatum, GPi and STN of BGS.

In this paper, according to the size of a rat brain and relative positional relationship of the STN, GPi and striatum of BGS, a novel dual-sided microelectrode array was designed and fabricated. Firstly, the fabricated microelectrode array was specially designed for studying the mechanisms of DBS in a rat PD model, which could stimulate STN and record discharge signals from the striatum, GPi and STN of BGS. Secondly, conventional single-side arrays may shield spike activity of some neurons facing the other side of the array, so a dual-side electrode array was developed by placing separately stimulating/recording sites on each planar surface of the microelectrode, which increased the density of stimulating/recording sites in the region of interest by twofold. Moreover, due to the characteristics of high frequency and high stimulating intensity of DBS, the diameter of stimulating sites (100 μm) is much larger than that of recording sites (20 μm), which would greatly enhance the charge injection capability of stimulating sites. Finally, the fabricated microelectrode arrays were characterized by scanning electron microscopy (SEM), electrochemical impedance spectroscopy (EIS) and other electrochemical methods. In addition, the stimulating effects of the fabricated microelectrode were evaluated by finite element modeling (FEM), and preliminary animal experiments showed that the designed microelectrode arrays could simultaneously record spontaneous discharge signals of multiple nuclei of BGS.

## 2. Materials and Methods

### 2.1. Microelectrode Arrays Design and Fabrication

**Figure 1:**
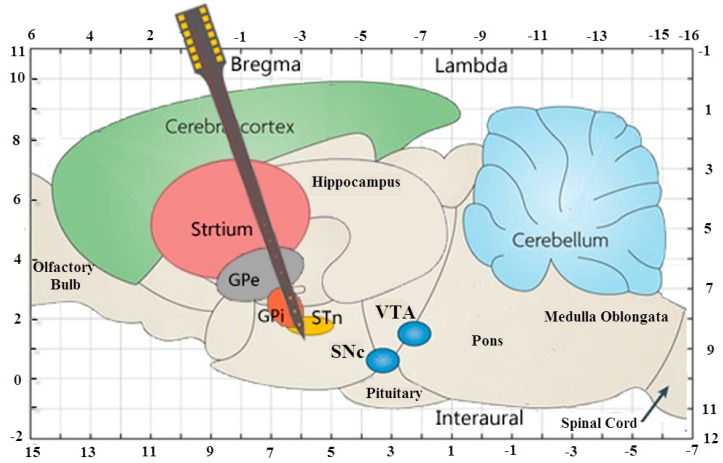
Schematic diagram of median sagittal plane of Sprague-Dawley rat brains showing the relative positional relationship of the STN, GPi and striatum (coordinate unit: mm).

**Figure 2:**
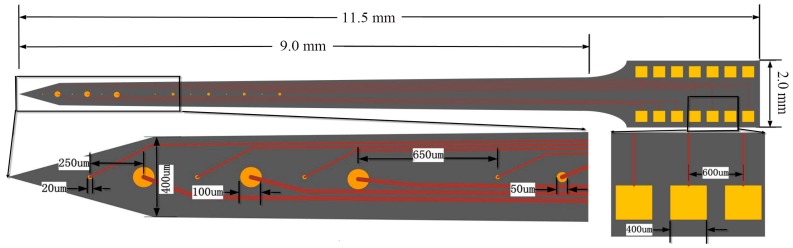
Schematic diagram of the designed microelectrode array.

The microelectrode arrays were designed according to the size of rat brain and relative positional relationship of the STN, GPi and striatum of BGS (Figure 1), and our purpose was to simultaneously record electrophysiological information from the striatum, GPi and STN of BGS. Figure 2 shows the detailed design parameters. The designed microelectrode array with dimensions of 9 mm × 400 μm × 200 μm (l × w × t) employed 14 round-shaped gold microelectrode sites distributed on each side. These sites could be divided into three types: three stimulating sites (diameter of 100 μm), seven electrophysiological recording sites (diameter of 20 μm) and four sites with diameter of 50 μm used for other experimental purposes in the future. For example, these 50 μm sites could be modified with the ion-exchange resin Nafion for detection of neurotransmitter dopamine [49], or modified with a protein matrix layer containing l-glutamate oxidase as glutamate sensors [50]. At the front of the designed microelectrode, three stimulating sites and three recording sites were mutually spaced with equal center-to-center distance of 250 μm. The distance between the tip and the center of the first recording site was 300 μm, defined by the fabrication process. These four sites with diameter of 50 μm and four recording sites were also mutually spaced with equal center-to-center distance of 250 μm. Each recording site was electrically connected to a bonding pad (400 μm × 400 μm) via 5 μm-wide gold conductive paths with 5 μm-wide gaps between them, and each stimulating site was electrically connected to a bonding pad via 10 μm-wide gold conductive paths with 5 μm-wide gaps between them.

**Figure 3:**
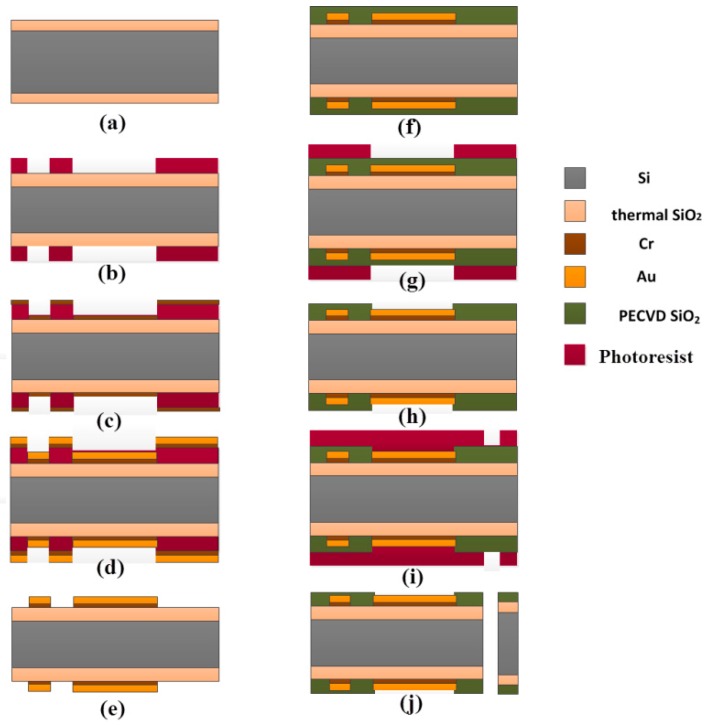
Schematic diagram of the process flow: (**a**) thermal oxidation; (**b**) spinning photoresist and patterning by lithography; (**c**) deposition of adhesion layer Cr; (**d**) deposition of Au layer; (**e**) lifting off the unpatterned metal and photoresist; (**f**) deposition of dielectric PECVD SiO_2_ layers; (**g**) spinning photoresist for patterning passivation layer; (**h**) exposing electrode sites and bonding pads; (**i**) spinning photoresist for DRIE; (**j**) DRIE and device release.

**Figure 4:**
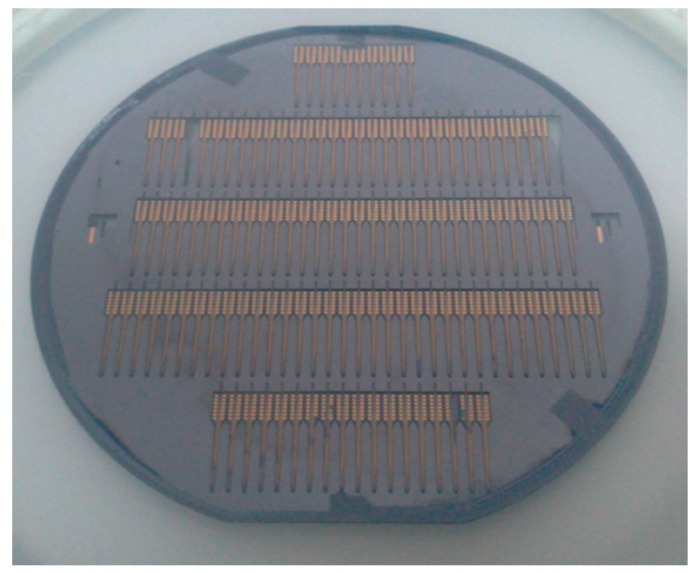
Picture of the entire wafer with 120 microelectrode array in holder frames.

Figure 3 showed the main fabrication process for a silicon-based microelectrode array. The substrate was typically comprised of about 100 mm diameter, double-side polished, p-doped (1 0 0) silicon wafers with a thickness of 200 μm. In the first step, the substrate was thermally oxidized at 1150 °C to yield a 1.5 μm silicon dioxide (SiO_2_) on both sides as an insulating layer (Figure 3a). Secondly, a 5 μm-thick negative tone photoresist (nLOF-2035, AZ Electronic Materials) was spun on and patterned to define the metal layer comprising the electrode sites, interconnecting wires and bonding pads by lithography (Figure 3b). This was followed by sputtering 20 nm-thick chromium (Cr) as an adhesion layer (Figure 3c) and evaporating a 300 nm-thick gold (Au) layer (Figure 3d) sequentially. The lift-off process was used to lift off the unpatterned metal and photoresist by dissolving the photoresist in an amine-solvent mixture (Figure 3e). Thirdly, the subsequent 2 μm-thick SiO_2_ passivation layer was made from plasma-enhanced chemical vapor deposition (PECVD) (Figure 3f). This passivation layer was patterned using reactive ion etching (RIE) through the photoresist as a hard mask (Figure 3g) to open electrode sites and bonding pads (Figure 3h). After the process on the front side, the wafer was turned over to repeat the above steps in the back. After that, additional twice lithography, left-off and RIE steps defined the dimensions of probes to realize the outline from the front and back surface of wafer by removing SiO_2_ outside the probe. Finally, a 10 μm-thick photoresist (AZ-9260, AZ Electronic Materials) was applied to serve as the etch mask layer (Figure 3i) and deep reactive ion etching (DRIE) was used to release the probes from the wafer (Figure 3j). The overview of an entire silicon wafer with the probes and frames is shown in Figure 4.

### 2.2. Microelectrode Arrays Package

**Figure 5:**
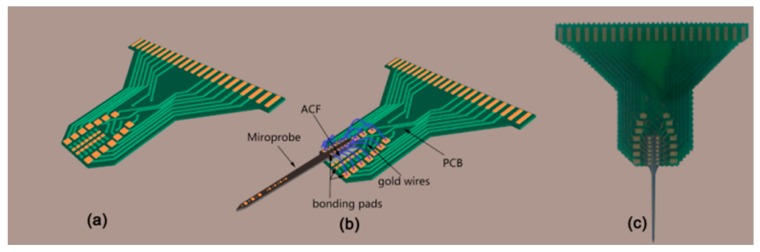
(**a**) Schematic diagram of the specifically designed PCB; (**b**) assembly scheme for connecting the double-sided microelectrode array with PCB; (**c**) optical image of schemed microelectrode array.

After fabrication of the microelectrode array, package was begun by combining gold wire bonding technology and flip-chip bonding technology, and a printed circuit board (PCB) specifically designed for this purpose was used for packaging (Figure 5a). On the back side of the microelectrode array, bonding pads between the microelectrode array and PCB were closely integrated by flip-chip technology, and the bonds were formed via an anisotropic conductive adhesive film (ACF, AC-7206U-18, HITACH): first, solder bumps were formed on inner bonding pads of PCB by reflow soldering, then a piece of ACF with a size of 1.5 × 5 mm completely covered every inner bonding pads of PCB by a temporary bonding at 80 °C, 1 Mpa for 5 s, and a final bonding was performed between the bonding pads of microelectrode array and ACF at 170 °C, 3 Mpa for 20 s. One the front side, 33 μm diameter gold wires connected the bond pads with traces on the PCB using gold wire bonding technology (Figure 5b,c). Additionally, a biocompatible adhesive was employed to strengthen the bond and seal the contact pads.

### 2.3. FEM of STN Stimulation

It would be difficult to perform experiments to directly measure the electric field distribution in brain tissue generated by DBS, but FEM provides a solution to quantitatively evaluate the neural response to DBS in a simulated environment. Three-dimensional models of STN stimulation were built in order to demonstrate the effects of extracellular stimulation using self-designed microelectrode array. The experiments could be mainly described in four steps: (1) a symmetric FEM of a neural probe surrounded by a homogeneous and isotropic volume conductor representing brain tissue was created using the FEM software COMSOL Multiphysics [51]; (2) the stimulus current was applied to the electrodes using a monopole stimulation mode at single or double side (reference electrode was at infinity for monopole stimulation mode); (3) the potential distribution in the model was calculated using a FEM solver; (4) and the volume of tissue activity (VTA) was estimated using MATLAB based on the extracellular excitation threshold of −0.5 V [52]. The electrical conductivity of electrode contacts and the substrate of the microelectrode array were 5.99 × 10^7^ and 1 × 10^−19^ S/m respectively [53], and an electrical conductivity of 0.2 S/m was applied in brain tissue [54]. Besides, we named the three stimulating sites #0, #1, #2, respectively, from the bottom to the top of the microelectrode array. The *X*, *Y* and *Z* axes were defined as follows: the Z axis was along the shaft of microelectrode probe (longitudinal axis of probe), the *Y* axis was vertical to the side surface of the probe, and the *X* axis was vertical to the *X*–*Y* plane.

### 2.4. Electrochemical Characterization of the Microelectrode Array

Electrochemical characterization of the microelectrode arrays were analyzed with the CHI 650E electrochemical analyzer (Shanghai CH Instruments, Shanghai, China) at room temperature in a conventional three-electrode electrochemical cell, which was composed of a fabricated microelectrode array as the working electrode, a platinum wire as the auxiliary electrode, and an Ag/AgCl as the reference electrode. The electrochemical impedance spectroscopy (EIS) was performed in 0.1 M phosphate buffer solution (PBS) at an amplitude of 50 mV and frequencies between 1 Hz and 100 kHz.

Here, electrochemical characterization of stimulating sites (diameter of 100 μm) and recording sites (diameter of 20 μm) were mainly studied. Before all the experiments, the microelectrode sites were pretreated in 0.5 M H_2_SO_4_ solution for cleaning surface of Au films until the cyclic voltammograms of Au microelectrode sites showed stable electrochemical behavior. In addition, in order to reduce the impedances of microelectrode sites, gold nanoparticles (AuNPs) were electrodeposited on the surface of microelectrode sites by immersing the microelectrode array into 0.1 M PBS (pH = 5.0) containing 1 mM chloroauric acid and applying a constant potential of −0.8 V for 200 s [55]. And then, the cyclic voltammograms of stimulating and recording sites at different scan rate (20, 50, 100, 150 and 200 mV/s) were obtained in 0.1 M KCl solution containing 2 mM [Fe(CN)_6_]^3−/4−^ (1 mM K_3_[Fe(CN)_6_] + 1 mM K_4_[Fe(CN)_6_]).

## 3. Results and Discussion

### 3.1. Morphological Characterization

**Figure 6:**
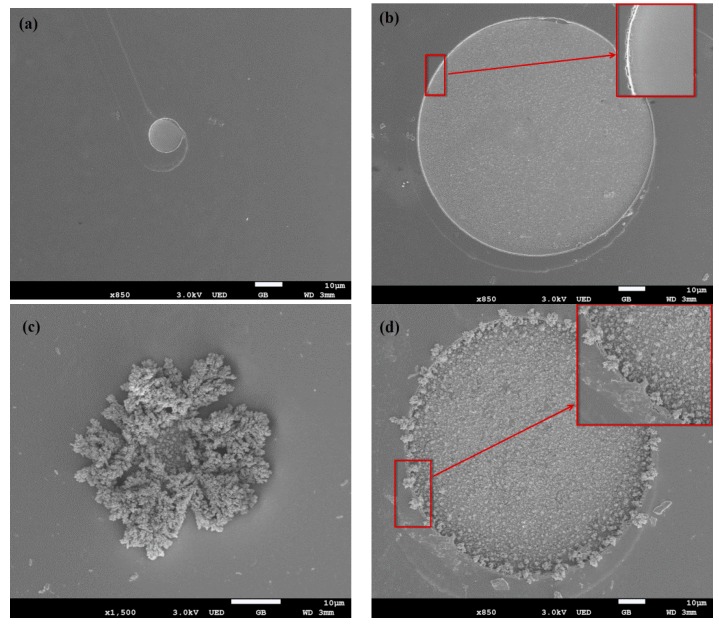
SEM images of stimulating sites (**b**,**d**) and recording sites (**a**,**c**) before (**a**,**b**) and after (**c**,**d**) electrodeposition of AuNPs.

**Figure 7:**
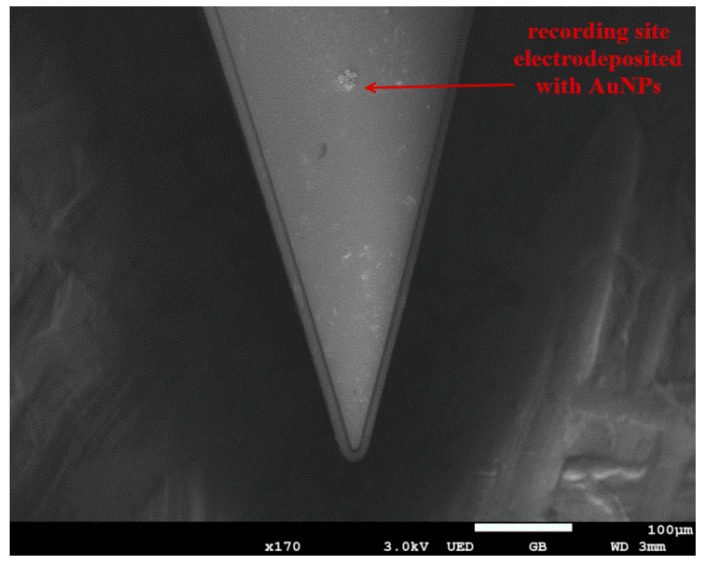
SEM images of tip of microelectrode shank.

The surface morphologies of the fabricated microelectrode array were examined by scanning electron microscopy (SEM), and here we mainly studied the morphological characterization of stimulating sites (diameter of 100 μm) and recording sites (diameter of 20 μm). As shown in Figure 6a, the recording site with a diameter of 20 μm possessed a round shape and smooth surface. Moreover, 5 μm-wide interconnecting wire connected with the sites was also visible. After electrodeposition of AuNPs (Figure 6c), dendritic nanoparticles with a 3D structure grown on the surface of the recording site, which could greatly increase the surface roughness, reduced the impedance of the electrode site and facilitated neural recordings. Figure 6b shows that the stimulating site with a diameter of 100 μm also had a round shape and smooth surface, and the inset figure with higher magnification indicates that there was a clear boundary between the gold layer and thermally oxidized SiO_2_, which illustrated that the passivation layer was well removed by reactive ion etching (RIE). After electrodeposition of AuNPs (Figure 6d), lots of gold nanoparticles were densely and uniformly distributed on the surface of the stimulating site, which greatly reduced impedance, increased surface roughness and enhanced the charge injection capability of stimulating sites [56]. Figure 7 showed an SEM image of the tip of a microelectrode array and a recording site located at the tip of the electrode shank. It could be clearly observed that a sharp tip geometry with accurate shape was presented, indicating that the microelectrode arrays were well released from silicon wafer by deep reactive ion etching (DRIE).

### 3.2. Effective Areas of STN Stimulation Based on FEM

**Figure 8:**
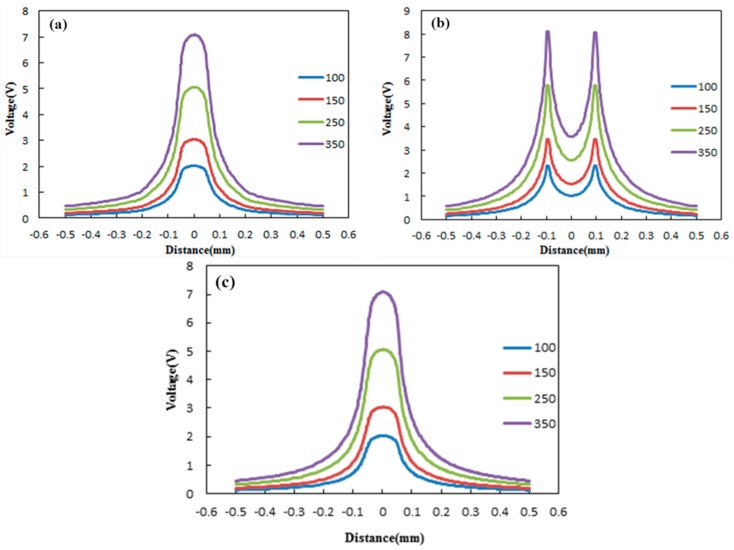
One-dimensional distributions of the extracellular potential (V) in three directions of *X*-axis (**a**); *Y*-axis (**b**) and *Z*-axis (**c**) generated by monopole stimulation in STN at various stimulus intensities (100, 150, 250 and 350 μA).

**Figure 9:**
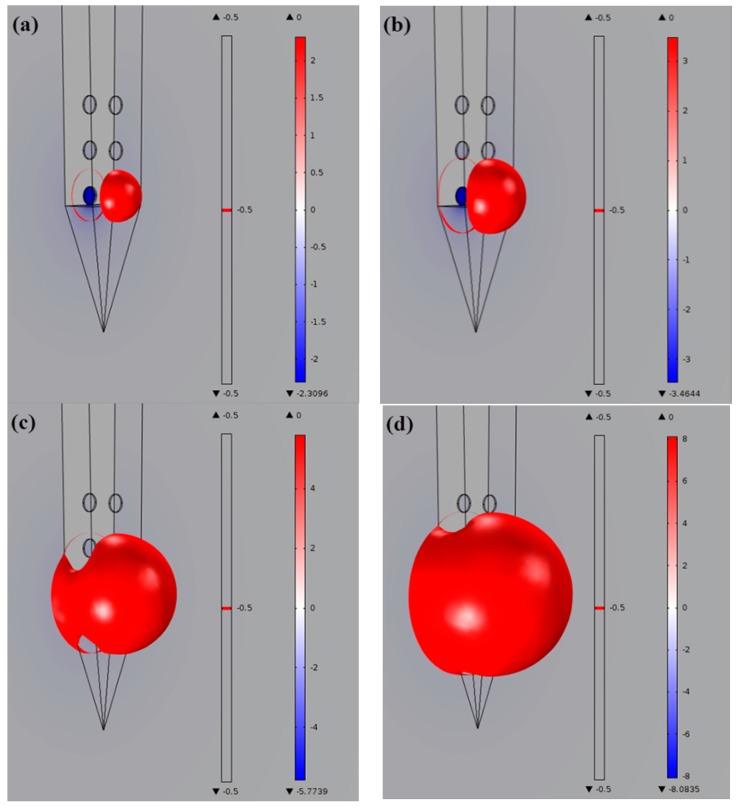
Three-dimensional extracellular potential distribution isosurface based on the extracellular excitation threshold of −0.5 V generated by monopole stimulation in STN at various stimulus currents (−100, −150, −250 and −350 μA).

**Figure 10:**
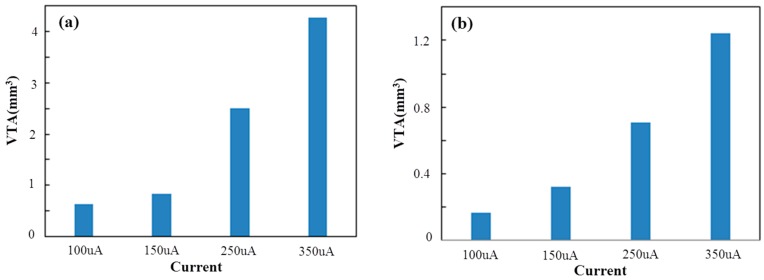
Effects of the stimulus intensity on the VTA during (**a**) double-side monopole stimulation; (**b**) single-side monopole stimulation.

The aim of this FEM simulation was to demonstrate the efficient spread of electrical stimulation in STN generated by DBS [57]. The extracellular potential (V) distributions in STN during monopole stimulation on double-side site #0 were studied. The extracellular potential (V) distributions generated from monopole stimulation at different current intensities in STN are shown in Figure 8 and Figure 9. Figure 8 shows the one-dimensional distributions of the extracellular potential (V) in three directions at various stimulus intensities. Figure 9 shows the three-dimensional extracellular potential distribution isosurface of −0.5 V (the extracellular excitation threshold) generated by DBS at different currents. When applying current intensities of 100, 150, 250 and 350 μA, the voltage values at the surface of electrode site #0 were 2.3096, 3.4644, 5.7739 and 8.0835 V, respectively. The potential distribution increased as the current stimulus was increased. In order to qualify the effects of the stimulation, VTA was calculated as the vital standard to measure the stimulus effects [58]. As shown in Figure 10a, when applying current intensities of 100, 150, 250 and 350 μA to STN, the VTA values were 0.6321, 0.8346, 2.5067 and 4.2805 mm^3^, which indicated that the VTA values increased with the stimulation intensities. Furthermore, we compared the stimulus effects of double-side monopole stimulation with that of single-side monopole stimulation. For single-side monopole stimulation, when applying current intensities of 100, 150, 250 and 350 μA, the VTA values were 0.1669, 0.3233, 0.7055 and 1.2404 mm^3^ (Figure 10b), showing that the VTA value of single-side monopole stimulation was significantly lower than that of double-side monopole stimulation (Figure 10a) when applying the same current intensity.

### 3.3. Electrochemical Behavior of the Microelectrode Array

**Figure 11:**
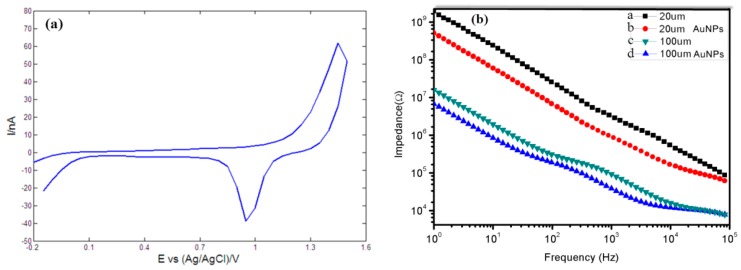
(**a**) Representative cyclic voltammogram of a recording site immersed in 0.5 M H_2_SO_4_; (**b**) impedances of a recording site (curve a, b) and a stimulating site (curve c, d).

Figure 11a shows the cyclic voltammetric response of a recording site with a diameter of 20 μm in 0.5 M H_2_SO_4_. The typical potential peak appearing at about 900 mV corresponded to the reduction of Au oxide species [59,60], implying that the cyclic voltammogram of the Au microelectrode site in H_2_SO_4_ matched the classic electrochemical response of bulk Au in H_2_SO_4_. These results indicated that thin film Au microelectrode sites possessed the same electrochemical properties as bulk Au and could be reasonably expected to possess the same recording capabilities as traditional Au microwires used for neural recording. In addition, the cyclic voltammetric responses of stimulating sites in H_2_SO_4_ possessed the same properties as those of recording sites.

The stimulating and recording sites were further electrochemically characterized by electrochemical impedance spectroscopy (EIS) to measure the electrode impedances in 0.1 M PBS using electrochemical analyzer and the results were presented in Figure 11b. For a typical recording site with diameter of 20 μm (curve a of Figure 11b) , the average measured impedance was about 4.1 MΩ at 1 kHz and the average impedance was reduced to about 0.9 MΩ after electrodeposition of AuNPs (curve b of Figure 11b), which was in rough agreement with previously published results [35,37]. The average impedance of a typical stimulating site with diameter of 100 μm reduced from about 200 KΩ (curve a of Figure 11c) to about 80 KΩ (curve a of Figure 11c) after AuNPs were deposited on its surface. These results implied that AuNPs could greatly increase electron transfer rates and surface roughness of electrode site, reduce interfacial impedances and enhance the performance of stimulating/recording sites.

**Figure 12:**
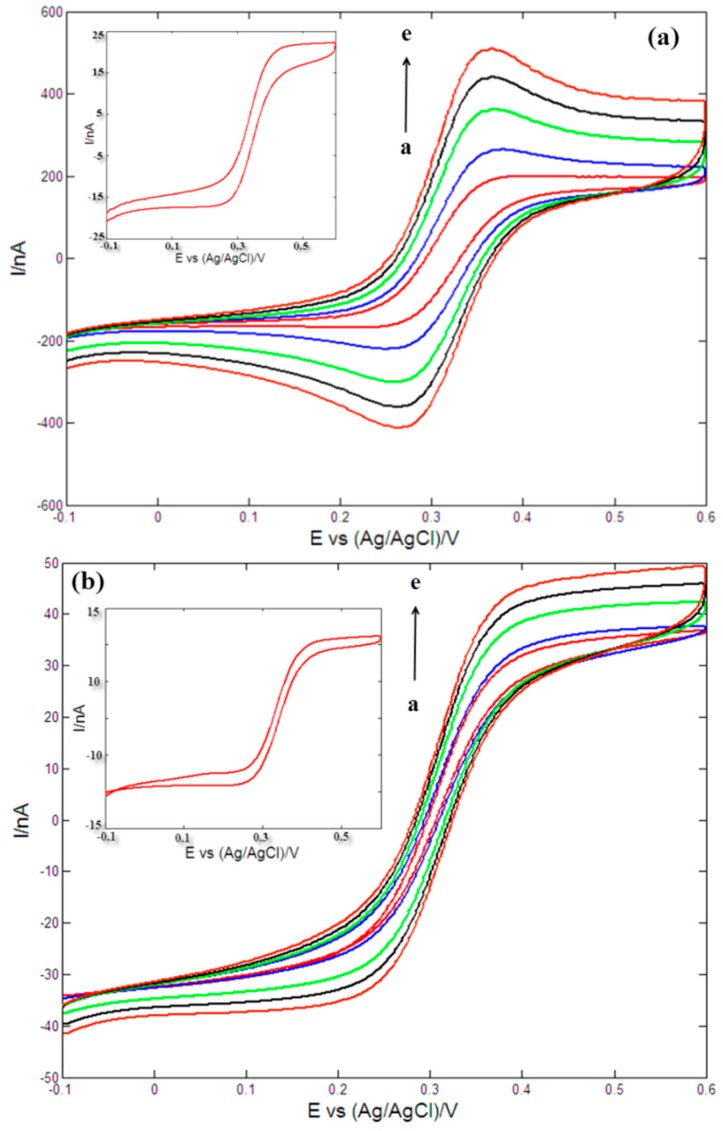
Cyclic voltammograms of the stimulating site modified with AuNPs (**a**) and recording site modified with AuNPs (**b**) in 0.1 M KCl solution containing 2 mM [Fe(CN)_6_]^3−/4−^. Scan rate: 20, 50, 100, 150 and 200 mV/s (from a to e). Inset of Figure 12a: cyclic voltammogram of bare stimulating site in 2 mM [Fe(CN)_6_]^3−/4−^ at scan rate of 100 mV/s. Inset of Figure 12b: cyclic voltammogram of bare recording site in 2 mM [Fe(CN)_6_]^3−/4−^ at scan rate of 100 mV/s.

The stimulating and recording sites modified with AuNPs were further characterized by measuring cyclic voltammograms in 0.1 M KCl solution containing 2 mM [Fe(CN)_6_]^3−/4−^ and the results were shown in Figure 12. As shown in Figure 12, the absolute values of reduction and oxidation peak currents were almost equal, and the peak potentials were definite values and independent of the scan rates, which indicated that the electrochemical processes of microelectrode sites in 0.1 M KCl solution containing 2 mM [Fe(CN)_6_]^3−/4−^ were reversible. For stimulating site (Figure 12a), the values of the peak currents increased with scan rates varying from 20 mV/s to 200 mV/s, and reduction and oxidation peak currents of the stimulating sites were proportional to the square root of the scan rates in the range 20–200 mV/s through analysis, suggesting the electrochemical responses were a typical surface-controlled process [61]. For the recoding site (Figure 12b), the cyclic voltammograms a typical showed a typical sigmoidal pattern due to the small area of the recording sites. The increase of peak currents was quite small with the increase of scan rates in Figure 12b due to the small size and edge effect of the recording sites [62]. These results demonstrated that the fabricated microelectrode array possessed good electrochemical properties and stability. In addition, inset of Figure 12a was the cyclic voltammogram of the bare stimulating site in 2 mM [Fe(CN)_6_]^3−/4−^ at scan rate of 100 mV/s, and the absolute values of reduction and oxidation peak currents of stimulating site modified with AuNPs (curve c of Figure 12a, scan rate of 100 mV/s) were much higher than those of the bare stimulating site (inset of Figure 12a) under the same experimental conditions, showing that AuNPs could greatly increase electron transfer rates and surface roughness of the electrode site. The same conclusion can be reached from the inset in Figure 12b.

### 3.4. In Vivo Neural Recordings

*In vivo* recordings in the brain of the anesthetized rats were performed to confirm the ability to record spontaneous discharge signals from multiple nuclei at the fabricated microelectrode array. Male Sprague-Dawley (SD) rats weighing 250–300 g (Laboratory Animal Center at Xi’an Jiaotong University School of Medicine) were used in the electrophysiological experiments. All animal experiments were conducted in accordance with protocols approved by the animal ethics committee of Xi’an Jiaotong University. The SD rats were anesthetized using an intraperitoneal injection of 1% pelltobarbitalum natricum (3 mg/100 g). After craniotomy and resection of the dura mater, the cortical surface was exposed. The microelectrode array was obliquely inserted into the rat brain at an angle of 72 degree with the horizontal plane from the pia mater (coordinates: 0 mm posterior and 2.5 mm lateral to Bregma and 0 mm subdural) by a micromanipulator with a constant speed of 10 μm/s and an insertion depth of 8 mm. Neural signals from the brain were recorded using a Cerebus multi-channel data acquisition system (Blackrock Microsystems, Salt Lake city, UT, USA). The original neural signals were amplified, filtered by a bandpass ranging from 1 Hz to 7.5 kHz and digitized (sampling rate: 30 kHz) for offline analysis.

**Figure 13:**
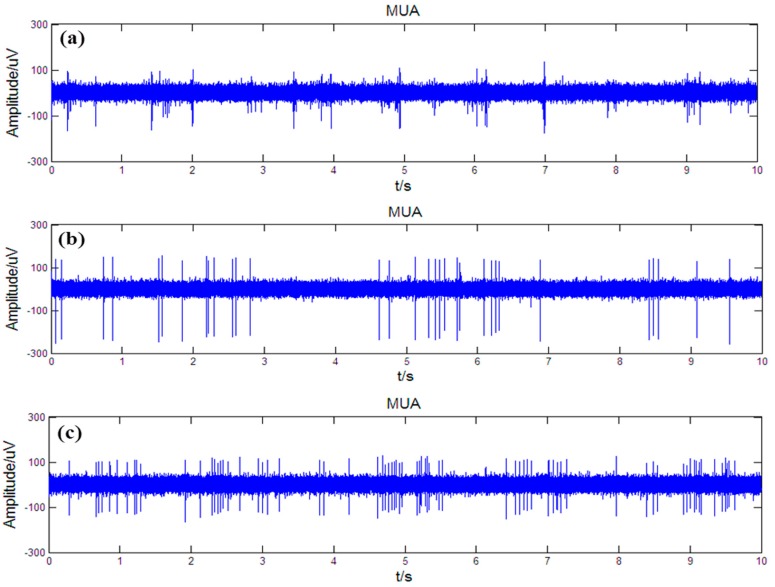
Simultaneously recorded MUA from striatum (**a**); GPi (**b**) and STN (**c**).

The obtained original neural signals were filtered by a bandpass from 500 Hz to 5 kHz to get the multiple unit activity (MUA) of nervous nuclei. Figure 13 showed the simultaneously recorded MUA from striatum, GPi and STN, which substantiated the ability of the fabricated microelectrode array to simultaneously record spontaneous discharge signals from multiple nuclei. In addition, the average signal-to-noise ratio (SNR) of the neural signals was calculated. The SNR of the neural signal was defined as the average peak-to-peak amplitude of spikes to the root mean square of the background noise, and the SNR of neural signals from the striatum, GPi and STN was 3.6, 6.3 and 4.7, respectively.

## 4. Conclusions

In this paper, the design, simulation, fabrication and characterization of a novel dual-sided microelectrode array have been reported. The microelectrode array was specially designed and fabricated for a rat Parkinson’s disease model to stimulate the subthalamic nucleus and simultaneously record electrophysiological signals from multiple nuclei of the basal ganglia system. The package of the dual-sided microelectrode array combined gold wire bonding technology and flip-chip bonding technology for the first time. In order to enhance the stimulating/recording functionality of the microelectrode, the impedances of the stimulating/recording sites were reduced by electroplating gold nanoparticles on the thin film gold electrode sites. SEM revealed that the stimulating/recording sites with accurate shapes and clear boundaries were well etched by reactive ion etching technology. *In vivo* neural recording experiments indicated that the designed and fabricated microelectrode array could successfully produce multiple-unit electrical activity from the striatum, GPi and STN simultaneously, demonstrating its potential application for studying the mechanisms of deep brain stimulation in rat models. Future work should concentrate first and foremost on integration of additional functionalities beyond stimulation and recording, such as integration of biosensors on the microelectrode array shaft [63], which can measure changes in the concentration of neurotransmitters when stimulating brain nucleus.
